# Factors associated to stigma in mental health workers of Castilla y Leon. The role of burnout and work motivation

**DOI:** 10.1192/j.eurpsy.2022.1775

**Published:** 2022-09-01

**Authors:** J.M. Pelayo-Terán, Y. Zapico-Merayo, M.E. Garcia Llamas, S. Vega-García, C. López-Zapico, M.R. Villa-Carcedo, Á. Álvaro-Prieto

**Affiliations:** 1 Hospital El Bierzo. GASBI. SACYL, Psiquiatría Y Salud Mental, Ponferrada, Spain; 2 Universidad Juan Carlos I, Facultad De Ciencias Jurídicas Y Sociales, Móstoles (Madrid), Spain; 3 Gerencia Regional de Salud de Castilla y León (SACYL), Serv. De Coord. Asis. Sociosanitaria Y Salud Mental, Valladolid, Spain

**Keywords:** stigma, burnout, humanization, mental health workers

## Abstract

**Introduction:**

Stigma is one of the most important barriers to help-seeking, treating maintenance and recovery for people suffering mental disorders. These attitudes, when present in mental health workers, may have a negative effect on the quality of health care.

**Objectives:**

to evaluate the levels of stigma in a representative sample of mental health workers and to explore potential modifiable factors associated to stigma attitudes.

**Methods:**

An online survey was conducted on the mental health workers of Castilla y León (Spain, 2409164 habs) while projecting the *2022 Mental Health Humanization plan* in order to asses educational skills, burnout (Maschlach MBI), Professional Quiality of life (CVP-35) and Stigma attitudes (Mental Illness: Cinician’s Attitudes Scales, MICA4) together with sociodemographic and work position variables.

**Results:**

193 workers completed completed the survey. Stigma Attitude values of the sample were low (MICA4: 31.71; SD:7.3) and burnout were low or medium (medium Emotional Exhaustion: 19.22; SD8.89; low Depersonalization: 4.91; SD:3.61; Medium Personal Accomplishment: 34,17; 6.3). In the linear regression (R2=0.249; F:11,527; p<0,001), a lower Stigma was predicted by psychologist (Beta:0,207; p=0,003) or psychiatrist position (Beta:0,204; 0,005), Self-efficacy assessed by the item “I am qualified” in the CVP-35 (Beta:-10,144; p=0,023), and a higher stigma was predicted by nurse assistant position (Beta: -0.230; p=0.001), Depersonalization Burnout dimension (Beta:0,351; p<0,001) and years of service (Beta:0.148; p=0,023)

**Figure:**
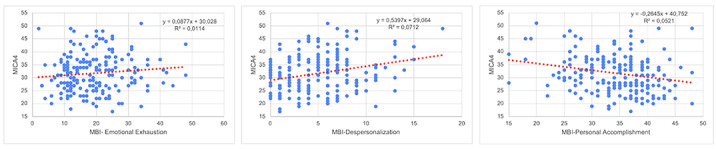

**Figure:**
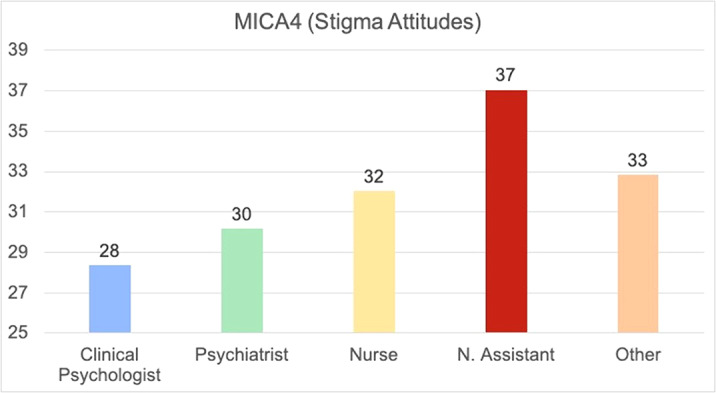

**Conclusions:**

Some groups of mental workers are more vulnerable to develop stigma attitudes. These, may be increased by fatigue and burnout. Future interventions should determine if reducing burnout and increasing capacitation may be effective in stigma eradication

**Disclosure:**

No significant relationships.

